# Keratin Biomembranes as a Model for Studying Onychomycosis

**DOI:** 10.3390/ijms21103512

**Published:** 2020-05-15

**Authors:** Anton Valkov, Michael Zinigrad, Alexander Sobolev, Marina Nisnevitch

**Affiliations:** Department of Chemical Engineering, Ariel University, Kyriat-ha-Mada, Ariel 4070000, Israel; antonva@ariel.ac.il (A.V.); zinigradm@ariel.ac.il (M.Z.); sobolev@ariel.ac.il (A.S.)

**Keywords:** keratin films, onychomycosis model, *Trichophyton rubrum*

## Abstract

Difficulties in obtaining human nails that are large enough for examining the penetration of drug formulations led us to produce keratin films regenerated from human hair. We assume that these films can simulate human nail plates in drug penetration and permeation tests and can serve as a biological model for studying onychomycosis. The films were formed from keratin extracted from human hair using dithiothreitol, urea and thiourea. The obtained keratin extract was dispensed into Teflon rings and dried at 40 °C and then cured at 110 °C. The structure, surface morphology, chemical characterization and thermal stability of the films were characterized and were compared to those of human nail, hair and bovine hoof samples using SDS-electrophoresis, scanning electron microscopy (SEM), X-ray diffraction analysis (XRD), Fourier transform infrared spectroscopy (FTIR) and thermogravimetric analysis (TGA). The structure of the obtained films was found to be closer to human nails than to hair or bovine hooves. The keratin films were infected with *Trichophyton rubrum* and were proven to be appropriate for serving as a model for studying onychomycosis.

## 1. Introduction

More than 10% of the global population is affected by fungal diseases on nail plates and nailbeds, also known as onychomycosis, which are caused mainly by dermatophytes [1]. No effective medication of onychomycosis has been proposed to date. Current treatment methods include local and oral drug delivery. Local treatment is preferable, since it has fewer negative impacts on internal organs and can be applied in situ. However, it also has some limitations caused by structural and physical properties of the nail plate, which serves as a barrier to the efficient transport of drugs to the infected site [2]. A high rate of drug penetration through the nail plate is a necessary but insufficient condition for successful topical treatment of fungal infections. The total amount of active components needed for effective treatment must also be taken into account [2]. 

Various drug formulations are used for enhancing drug transport through nail plates, including transfersomes, nanoemulsions, gels, creams, and ointments for topical application [1,3]. Since the drug delivery rate affects its efficiency against fungal infection, choosing a suitable drug formulation can be critical for the success of the treatment. For instance, lipophilic drugs are delivered to fungal colonies faster than hydrogel ointments. The effectiveness of the antifungal drugs can be evaluated in ex vivo experiments using human nail plates. However, such studies are limited due to the low availability of whole human nail plates. Ex vivo experiments using bovine hoof plates instead of nail plates are often performed, but their results do not demonstrate the actual effectiveness of the tested drugs in humans [4].

Most antifungal drugs are lipophilic and insoluble in water. In some tests for antifungal activity (i.e., Clinical Laboratory Standards Institute, CLSI), these drugs are dissolved in dimethyl sulfoxide. Surfactants can be added to solubilize the antifungal drugs. The prepared solutions are usually injected into liquid or solid media that are pre-infected with fungi or their spores, and the efficiency of the antifungal agents is tested only qualitatively. Therefore, this method cannot be considered universal, since it does not relate to nail involvement and microstructure.

Another approach to testing antifungal drugs in in vitro experiments is based on adding crushed pieces of human nail plates to the medium as a source of keratin [5,6]. It has been shown that the minimal inhibitory concentration (MIC) of the tested antifungal drugs significantly increased after adding the nail powder, which indicates the necessity of the presence of keratin in antifungal experiments. Osborne et al. [5] emphasized the importance of analyzing the antifungal activity of drugs on natural substrates, i.e., using human nail plates or keratin-based materials with a similar structure and properties, instead of media enriched with nutrients. It should also be noted that keratin serves not only as a nutrient for dermatophytes, but also plays an up-regulatory role for some virulence factors [7].

A model that can be used to evaluate the effectiveness of various drug formulations could make a significant contribution to preclinical and preliminary assessments of drug penetration. Such models have not been developed to date due to low availability of human nail material. For this reason, it is necessary to produce materials that have similar structural and physiological characteristics to human nail plates and can replace them with maximal similarity to in vivo tests.

Several models have been proposed for predicting transungual drug absorption into human nail plates [8,9]. However, these models require individual nail plates, which complicate their widespread use. Other models use membranes based on bovine hooves [10]. These models do not provide a close approximation to human nails, since the density of the keratin fibers matrix in bovine hooves is lower compared to the human nail. The low density of the matrix in bovine hooves is due to fewer disulfide bounds compared to human nail plates [11,12]. As a result, bovine hooves can be less sensitive to factors disrupting disulfide bonds applied to increase the permeability of drugs.

Lusiana et al. [13] proposed using keratin films made from human hair. This type of film is suitable for in vitro simulations. Sufficient size and flat shape of the prepared membranes enable the application of the drugs to the membrane surface in a uniform manner to study their penetration potential.

In the present work, keratin films were produced using keratin extracted from human hair. The structure of the films was compared to that of human nail plates and bovine hooves using X-ray diffraction analysis (XRD), scanning electron microscopy (SEM), thermogravimetric analysis (TGA) and Fourier transform infrared spectroscopy (FTIR). The aim of this study was to prepare keratin films for replacing nail plates in permeation and penetration studies of antifungal drugs. The keratin films can be used in further experiments on modelling and treatment of onychomycosis. 

## 2. Results and Discussion

### 2.1. Protein Extraction from Human Hair

**Figure 1:**
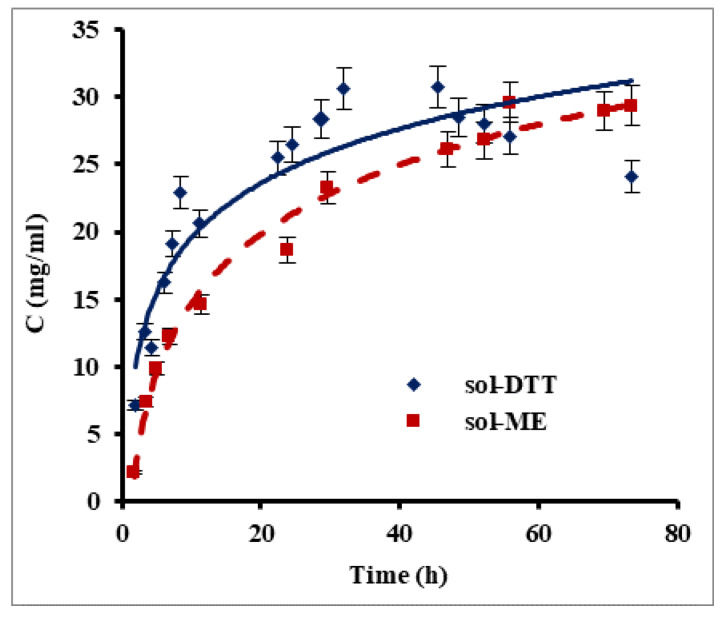
Protein extraction from human hair using β-mercaptoethanol (sol-ME) and 1,4-dithiothreitol (sol-DTT) containing solutions. Protein was extracted at 50 °C from delipidized human hair. Protein concentration was measured by the Bradford assay. Error bars present standard errors.

Keratin films were prepared using human hair as an available source of keratin. Since keratin is water insoluble, it was necessary to disrupt disulfide bonds for its extraction into an aqueous phase. For this purpose, we compared between two reagents which have good ability to reduce the disulfide bonds in proteins: β-mercaptoethanol and 1,4-dithiothreitol (DTT). Two extraction methods, one based on a β-mercaptoethanol-containing solution (sol-ME) (Shindai method) [14] and one using a 1,4-dithiothreitol-based solution (sol-DDT), were compared for their keratin extraction rate. Figure 1 presents changes in protein concentration during keratin extraction using the sol-ME and sol-DTT solutions. A maximal protein concentration of 30 mg mL^−1^ was obtained after ca. 32 h of extraction with the sol-DDT solution, whereas a protein concentration of 28 mg mL^−1^ was reached after 70 h with the sol-ME solution. During the first 7.5 h, the keratin extraction rate was significantly higher when using sol-DTT compared to sol-ME: 2.77 mg mL^−1^ h^−1^ and 1.84 mg mL^−1^ h^−1^, respectively (*p* value = 0.05). The solutions became opaque over the course of the keratin extraction, probably due to protein aggregation. This phenomenon may also explain an observed decrease in protein concentration in sol-DTT after 32 h. All subsequent extractions were therefore performed using the sol-DTT solution for 30 h. 

Nakamura et al. [14] previously reported that extracting solutions containing DTT or β-mercaptoethanol at equal concentrations were equally efficient in protein extraction. However, our experiments show that the extraction rate increased by 50% when using the sol-DTT extractant, even when the DTT concentration was much lower than that of β-mercaptoethanol. In both cases, the final concentration of extracted protein in our study was more than 15-fold higher than in the previous research [14].

### 2.2. SDS–PAGE Analysis of Keratin Extracts

**Figure 2:**
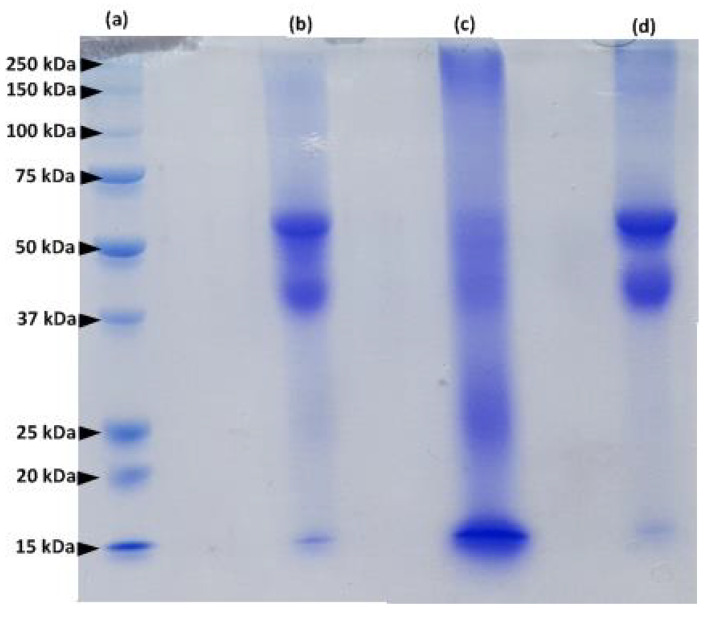
SDS–PAGE of a molecular weight markers (lane a); human nail extract (lane b); bovine hoof extract (lane c) and human hair extract (lane d).

The protein extracted from human hair was examined by sodium dodecyl sulfate polyacrylamide gel electrophoresis (SDS-PAGE) and compared to the protein extracted from human nails and bovine hooves (Figure 2). Protein fractions of hair and nail extracts are similar (lanes d and b in Figure 2). 

Major protein bands with a molecular mass between 40–60 kDa represent microfibril keratins, and the 15 kDa bands correspond to matrix proteins [14]. However, the profile of the hoof extract (lane c, Figure 2) is different from that of human hair and nails and contains additional protein bands: the major protein fragments are characterized by a MW of 15, 25, 40–60 and 250 kDa. These results are in accordance with previous studies of keratinized tissues [13,14,15,16]. Our findings confirm the similarity between proteins composing human hair and nails.

### 2.3. Preparation of Keratin Films

**Figure 3:**
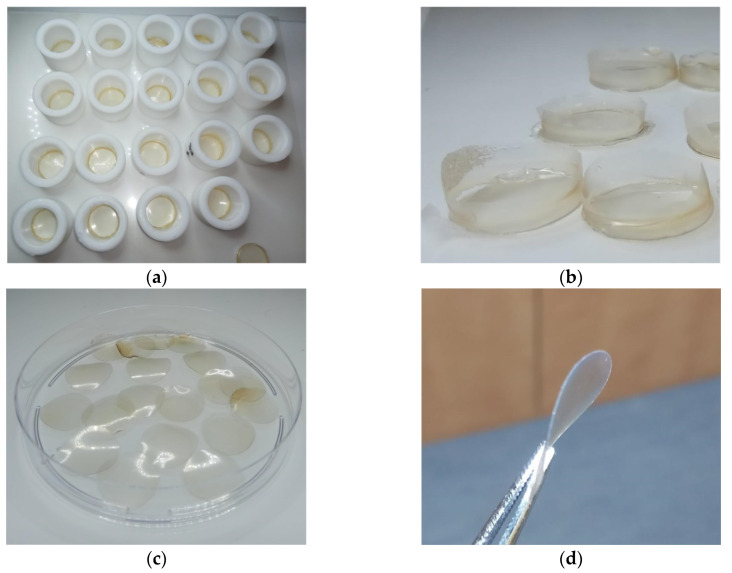
Keratin films: (**a**) Intermediate soft keratin films (KF-40) obtained in 20 mm Teflon rings; (**b**) KF-40 keratin films before punching; (**c**) 16 mm diameter KF-40 keratin films after punching; and (**d**) KF-110 keratin films after curing.

Extraction of keratin from human hair for preparation of keratin films was performed using the sol-DTT solution. The concentration of the extracted protein in the various batches ranged between 30–35 mg mL^−1^. The obtained extracts were filtrated and dialyzed. Keratin precipitation was observed during dialysis of the filtrates, accompanied by aggregation due to formation of cross-linking disulfide bonds. The aggregates were removed by centrifugation, and as a result, the protein concentration decreased to ca. 20 mg mL^−1^. For film formation, equal portions of this solution were dispensed into Teflon rings (Figure 3a) and excessive water was evaporated at 40 °C, yielding soft keratin films designated as KF-40 (Figure 3b). The keratin films were punched in order to get rid of uneven edges (Figure 3c). The films were then cured at 110 °C for additional evaporation of water. This led to the formation of rigid keratin films designated as KF-110 (Figure 3d). The width of the films was 67 ± 3 μm. 

### 2.4. X-ray Diffraction Analysis (XRD) of Keratin Films

**Figure 4:**
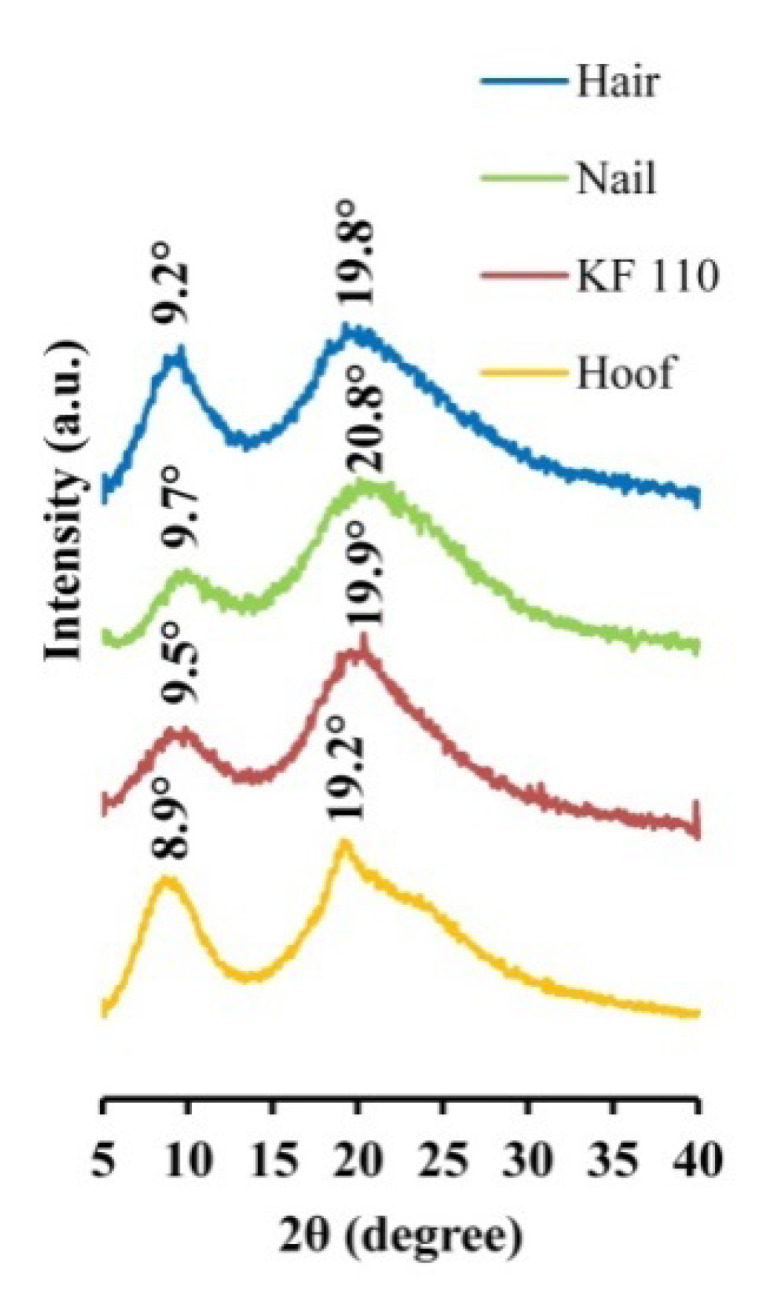
X-ray diffraction patterns of human hair, nail, KF-110 and bovine hoof samples.

The produced KF-110 films were examined by XRD and compared to human hair, nail and bovine hoof samples. The XRD patterns of the samples are presented in Figure 4. Two main bands characteristic of α-keratin 2 θ bands are present in all spectra: KF-110 yielded 9.5° and 19.9° bands, hair—9.2° and 19.8°, a nail sample—9.7° and 20.8°, and a hoof sample—8.9° and 19.2° bands. Minor shifting between the bands in our samples may indicate some differences between crystalline domains of the keratin. According to previous reports [17,18,19,20], α-keratin has 2 θ bands around 9.02–9.26°, corresponding to the distance between α-helical axes of 0.954–0.979 nm, and 17.2–20.6° which characterize the 0.431–0.515 nm distance to the α-helix pitch projection [19]. The XRD results show that the structure of α-keratin was preserved in the obtained keratin films after the processing treatments. It should be mentioned that the XRD profile of the KF-110 films was much closer to the nail pattern than to hair from which the films were produced or the hoof sample. These data indicate that the manufactured films have a nail-like structure and may serve as a nail model in further experiments. The hoof structure was found to be different from that of nails. Artificial films originating from human hair can therefore be considered as a better model for human nails than bovine hooves.

### 2.5. Fourier Transform Infrared Spectroscopy (FTIR) Analysis of Keratin Films

**Figure 5:**
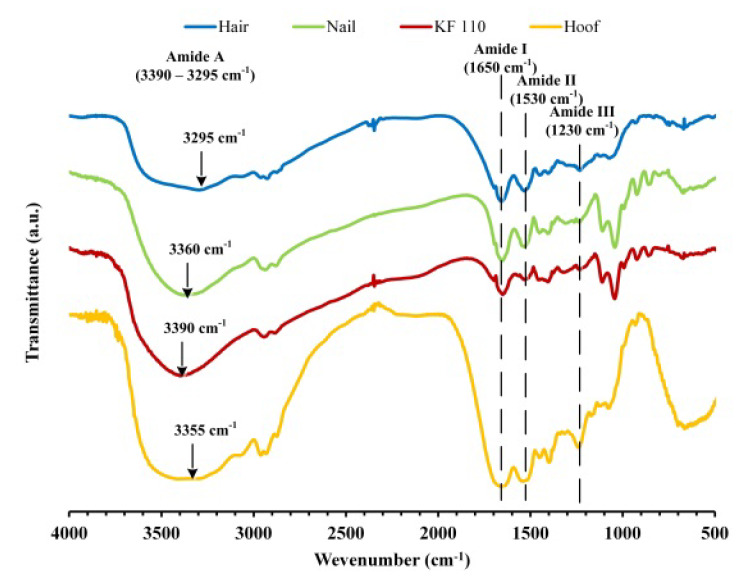
Fourier transform infrared spectroscopy (FTIR) spectra of human hair, nail, KF-110 and bovine hoof samples.

FTIR spectra of human hair, nail, KF-110 and bovine hoof samples were measured in order to analyze functional groups in keratin patterns (Figure 5). The spectra have characteristic absorbance bands of proteins that can be assigned to peptide bonds (–CONH–) known as the amide A (3000–3500 cm^−1^), amide I (1600–1700 cm^−1^), amide II (1510–1580 cm^−1^) and amide III (1220–1330 cm^−1^) peaks [21]. Broad absorbance peaks of amide A at 3295, 3360, 3390 and 3355 cm^−1^ in the spectra of human hair, nail, KF-110 and hoof samples, respectively, can be corresponded to N–H stretching vibrations characteristic for amide A. This mode of vibration does not depend on the sample backbone. However, it is very sensitive to the strength of hydrogen bonds, so that peak shifting can be explained by various rates of sample humidity. The remaining absorbance bands can be assigned to stretching vibrations of C=O and C–N groups (amide I, 1650 cm^−1^), N–H bending, C–N and C–C stretching vibrations (amide II, 1530 cm^−1^) and C–N stretching and C=O bending vibrations (amide III, 1230 cm^−1^) [22,23]. The FTIR analysis showed that the functional groups in the obtained keratin films remained similar to groups in the original hair. It should be mentioned that nails and KF-110 films have identical spectra (Figure 5, red and green curves). However, the spectra of hair and hoof samples differ from those of nails and KF-110 in the fingerprint region, since bands 1108, 1043, 990, 920 and 860 cm^−1^ characteristic for nails are absent. The qualitative and quantitative composition of functional groups in the obtained keratin films is probably very close to that of nails. This supports the assumption that keratin films can serve as a good model for nails.

### 2.6. Thermogravimetric Analysis (TGA) of Keratin Films Done

**Figure 6:**
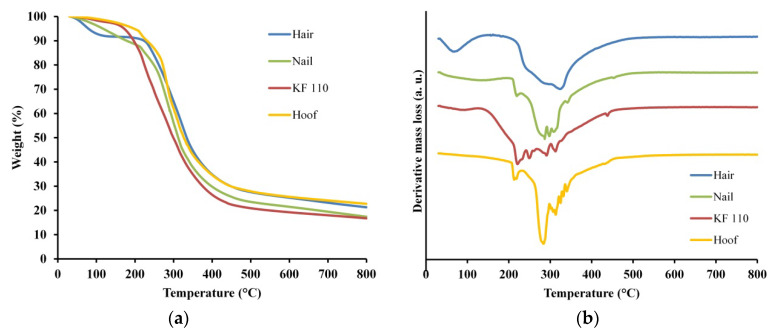
Thermogravimetric analysis (TGA) (**a**) and derivative thermogravimetry (DTG) (**b**) curves for human hair, nail, KF-110 and bovine hoof samples.

Thermal stability and degradation of human hair, nail, KF-110 and bovine hoof samples was studied using TGA. The TGA curves of the samples are presented in Figure 6a and the derivative thermogravimetry (DTG) graphs in Figure 6b. Two regions of major mass losses are observed in all curves, the first is up to 200 °C and the second is at 200–400 °C (Figure 6a). The first mass loss step was registered at temperatures up to 150 °C for KF-110 and up to 200 °C for hair, nails and hooves. This mass loss can be assigned to evaporation of incorporated water, including free water and loosely bonded water [24]. The difference in total water evaporation between KF-110 and other samples can be explained by the fact that the curing of KF-110 was performed at 110 °C and at this stage a major part of the water already evaporated. The second mass loss step of the samples was registered at about 400 °C. This mass loss can be related to degradation of the organized structures and denaturing of the keratin matrix [25,26,27]. The second and main region of mass loss can be explained by keratin decomposition and release of volatile compounds such as CO, H_2_S, CH_4_ and HCN, with maximum emission of these products at 420, 265, 580 and 345 °C, respectively [28]. Additionally, all decomposition processes are accompanied by additional water release. DTG curves of the examined samples show decomposition steps at 250, 290, 323 °C for hair, at 219, 286, 297, 307, 341, 453 °C for nails, at 220, 249, 290, 312, 438 °C for KF-110, and at 213, 284, 313, 325, 332, 340 °C for a hoof sample (Figure 6b). These data show that the decomposition profile of KF-110 partially resembles the profile of nails, although the KF-110 curve has an additional step at 249 °C (Figure 6b). At the same time, the curve for KF-110 differs from that of hair and hooves (Figure. 6b). The differences between the temperatures of decomposition steps can be explained by various types and sizes of keratin matrices and by the number of disulfide bonds, which directly depends on the number of cysteine residues in the proteins. Human hair has 1.3 times more cysteine residues than human nails [29,30,31], and this affects their decomposition profiles. We assume that the fact that the maximal decomposition temperature of KF-110 is lower than that of hair, which is the source material for the films, can be explained by incomplete formation of disulfide bridges in keratin molecules during the curing stage of KF-110 preparation.

### 2.7. Scanning Electron Microscopy (SEM) of Keratin Films

**Figure 7:**
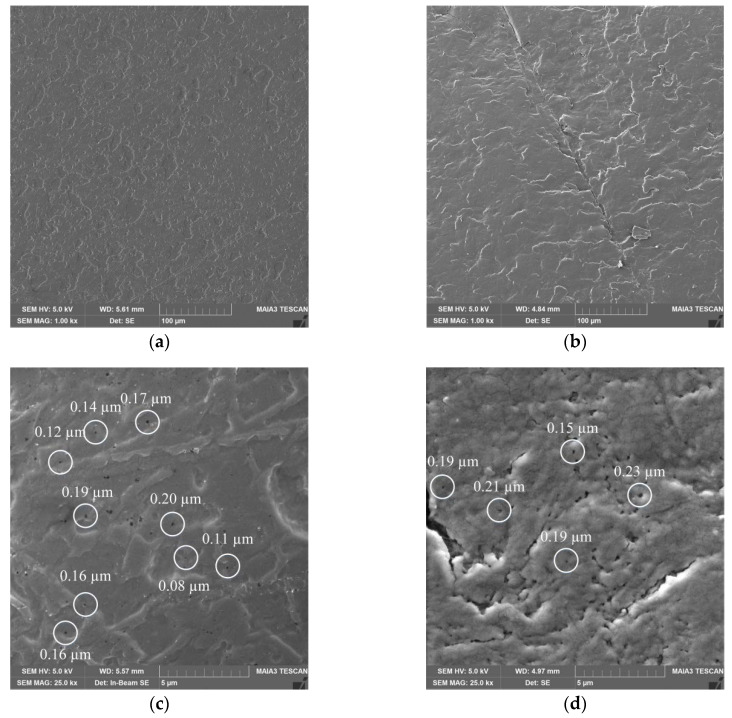
Scanning electron microscopy (SEM) micrographs of KF-110 films (**a**,**c**) and nail plates (**b**,**d**) at magnifications of 1000× (**a**,**b**) and 25,000× (**c**,**d**). White circles on (**c**) and (**d**) point on pores size of which was measured.

The obtained KF-110 and real nail surfaces were examined by SEM analysis. As can be seen in Figure 7, the KF-110 films and nail plates have a similar general morphology, although the former shows a denser and less layered structure (Figure 7a) compared to nail plates (Figure 7b). At a high magnification, it can be clearly seen that both samples have a porous structure with pore sizes ranging between 0.1–0.2 μm for KF-110 and 0.1–0.3 μm for the nail plates (Figure 7c,d, respectively).

### 2.8. Infection of Keratin Films by Trichophyton Rubrum 

**Figure 8:**
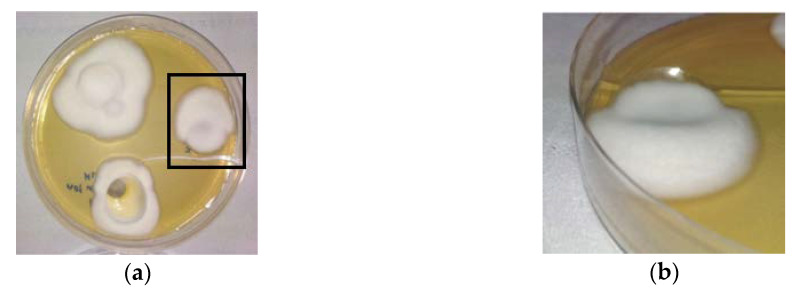
Keratin films infected by the dermatophyte *T. rubrum*: (**a**) a top view and (**b**) a side view of the film framed in Figure 8a.

Since a certain structural similarity was found between keratin films and nails, it was important to test whether the films could serve as an appropriate model for onychomycosis studies. For this purpose, the keratin films were infected with the dermatophytic fungus *Trichophyton rubrum* (*T. rubrum*). Three days after infection, all films were not only covered superficially by white and cotton-like fungi on the surface (Figure 8a), but fungi also penetrated inside and through the films (Figure 8b). These observations show that the obtained KF-110 films can be successfully infected with dermatophytes and can be used as a substitute for nails in in vitro experiments on onychomycosis.

## 3. Materials and Methods

### 3.1. Extraction of Protein from Human Hair

Human hair was obtained from a local hair salon with a customer permission and thoroughly washed. External lipids were removed using a mixture of chloroform and methanol (2:1, *v/v*) for 24 h. After removal of the solvents, the hair was dried in a chemical hood. 1 g of delipidized hair was mixed with 20 mL of Shindai solution (sol-ME) [14] containing 25 mM Tris.HCl, pH 8.5 (Merck, Kenilworth, NJ, USA), 2.6 M thiourea (Sigma-Aldrich, St. Louis, MO, USA), 5 M urea (Sigma-Aldrich, St. Louis, MO, USA) and 0.717 M β-mercaptoethanol (Merck, Kenilworth, NJ, USA) or of a modified Shindai solution (sol-DTT) containing 0.102 M 1,4-dithiothreitol (Merck, Kenilworth, NJ, USA) instead of β-mercaptoethanol. Extraction was performed for 72 h under shaking at 50 °C. Proteins were also extracted from human nails and bovine hooves using sol-DTT as described above. After extraction, the protein solution was filtered using medical gauze, dialyzed and centrifuged at 12,000× *g* for 30 min at 20 °C. The concentration of protein released from each sample was monitored by the colorimetric Bradford method [32] using the Bio-Rad protein assay reagent [33] and bovine serum albumin (Sigma-Aldrich, St. Louis, MO, USA) as a standard. 

### 3.2. Keratin Film Preparation

The extract obtained as described in Section 3.1 was dialyzed against deionized water using Cellu Sep T2 tubing of MWCO 6000–8000 Da (Membrane Filtration Products Inc., Seguin, TX, USA) for at least 48 h at the ambient temperature. The dialysate was then centrifuged at 12,000× *g* for 30 min at 20 °C to remove coarse aggregates using an Avanti j-E centrifuge (Beckman Coulter, Indianapolis, IN, USA). Glycerol (Merck, Kenilworth, NJ, USA) was added to the dialysate up to 1% (w/w) as a film plasticizer, and the mixture was stirred in a planetary centrifugal mixer Thinky ARE-250 (Thinky Corporation, Tokyo, Japan) for 2 min. 2 mL portions of this mixture were dispensed into polytetrafluoroethylene (Teflon) rings having a 2 cm inner diameter attached to a siliconized polyethylene terepthalate foil that served as a base. The rings were placed in a drying oven at 40 °C for 24 h. The produced intermediate soft films (KF-40) were punched out using a hollow punch tool with a 16 mm diameter. The KF-40 films were then cured between two metal plates at 110 °C for 3 h to enable re-formation of disulfide bridges between keratin molecules to produce stable regenerated keratin films (KF-110). The width of KF-110 was measured by Digital Outside Micrometer MT-211101 (Munro Instruments, Essex, UK).

### 3.3. SDS–PAGE Analysis

The molecular weight of the proteins extracted from human hair, nail and bovine hoof was determined by SDS-PAGE. The protein solutions were diluted to a concentration of 2 mg mL^−1^, mixed with Laemmli sample buffer (Bio-Rad, Hercules, CA, USA) and 5% 2-mercaptoethanol (Sigma-Aldrich, St. Louis, MO, USA), denatured by boiling for 5 min and cooled in ice. The protein samples were loaded onto a slab gel (4% stacking gel and 12.5% separating gel). Electrophoretic separation was performed on a Mini-protean Tetra System (Bio-Rad, Hercules, CA, USA) using the Precision Plus Protein Standard (Bio-Rad, Hercules, CA, USA). The gel was stained with Coomassie brilliant blue R-250 (Sigma-Aldrich, St. Louis, MO, USA) and de-stained with a de-staining solution (7% *v/v* acetic acid, 40% *v/v* methanol). 

### 3.4. XRD Analysis

The structure of human hair, nail, bovine hoof samples and KF-110 was examined by XRD analysis using a XPert PRO instrument (PANalytical, Almelo, Netherlands) with a Cu Kα radiation at 40 kV and 40 mA, with 2 θ ranging from 5° to 40° at a step size of 0.02° and 12 s scan step time.

### 3.5. FTIR Analysis

FTIR spectra of human hair, nail, bovine hoof and KF-110 were measured using a FTIR Spectrometer Spectrum One (Perkin Elmer, Waltham, MA, USA). The spectra were registered from 400 to 4000 cm^−1^ by 16 scans. The samples were mixed with KBr (Spectrum, Stamford, CT, USA) at a 1:100 (*w/w*) sample to KBr ratio and ground vigorously. The pellets were formed using a press (Equilab, Madrid, Spain) under 5000 kg_f_ for 2 min with the help of a 13 mm pellet die (Carver, Wabash, IN, USA).

### 3.6. TGA Analysis

TGA measurements of human hair, nail, bovine hoof and KF-110 were performed with the TGA/DSC 1 STARe System (Mettler Toledo, Columbus, OH, USA). 8–10 mg samples were analyzed in 70 µL alumina crucibles under a nitrogen atmosphere at a flow rate of 40 mL min^−1^, in a temperature range between 30 °C and 800 °C at a heating rate of 10 °C min^−1^.

### 3.7. SEM Analysis

The morphology of the keratin samples, human nails, bovine hoof and regenerated keratin film (KF-110) from human hair were investigated by a scanning electron microscope MAIA3 (TESCAN, Brno, Czech Republic) at an accelerating voltage of 30 kV. Prior to examination, surface samples were placed on an adhesive stub and coated with gold under a vacuum for 30 s using a Q150T ES coating system (Quorum Technologies LTD, Lewes, UK). 

### 3.8. Infection of Keratin Films

The infection of keratin films with dermatophytes was performed by preliminary incubation of KF-110 with a *Trichophyton rubrum* (*T. rubrum*, ATCC^®^ MYA-4438™) inoculum in YM broth (Difco™, Bergen County, NJ, USA) for 48 h at 30 °C. The films were then placed on YM agar (Difco™, Bergen County, NJ, USA) in Petri dishes for further incubation for 7 days at 30 °C.

### 3.9. Statistical Methods

The results of all experiments were obtained from at least three independent experiments carried out in duplicates and were statistically analyzed by ANOVA single factor analyses. The difference between the results was considered significant if the *p* value was less than 0.05. Error bars in the graphs of protein extraction from human hair present the standard errors (SE).

## 4. Conclusions

Keratin films prepared from human hair have a human nail like structure and can be used as a nail model for studying onychomycosis.

## Abbreviations

KF	Keratin film
DTT	1,4-Dithiothreitol
ME	β-Mercaptoethanol
FTIR	Fourier transform infrared spectroscopy
SDS–PAGE	Sodium dodecyl sulfate polyacrylamide gel electrophoresis
XRD	X-ray diffraction analysis
TGA	Thermogravimetric analysis
DTG	Derivative thermogravimetry
SEM	Scanning electron microscopy
